# In-Line Monitoring of Milk Lactose for Evaluating Metabolic and Physiological Status in Early-Lactation Dairy Cows

**DOI:** 10.3390/life15081204

**Published:** 2025-07-28

**Authors:** Akvilė Girdauskaitė, Samanta Arlauskaitė, Arūnas Rutkauskas, Karina Džermeikaitė, Justina Krištolaitytė, Mindaugas Televičius, Dovilė Malašauskienė, Lina Anskienė, Sigitas Japertas, Ramūnas Antanaitis

**Affiliations:** 1 Large Animal Clinic, Veterinary Academy, Lithuanian University of Health Sciences, Tilžės Str. 18, LT-47181 Kaunas, Lithuania; samanta.arlauskaite@lsmu.lt (S.A.); arunas.rutkauskas@lsmu.lt (A.R.); karina.dzermeikaite@lsmu.lt (K.D.); justina.kristolaityte@lsmu.lt (J.K.); mindaugas.televicius@lsmu.lt (M.T.); dovile.malasauskiene@lsmu.lt (D.M.); ramunas.antanaitis@lsmu.lt (R.A.); 2 Department of Animal Breeding, Faculty of Animal Sciences, Lithuanian University of Health Sciences, Tilžės 18, LT-47181 Kaunas, Lithuania; lina.anskiene@lsmu.lt; 3 Practical Training and Research Center, Lithuanian University of Health Sciences, Topolių g. 6, LT-54310 Kaunas, Lithuania; sigitas.japertas@lsmu.lt

**Keywords:** milk lactose, dairy cows, innovative technologies, precision dairy farming

## Abstract

Milk lactose concentration has been proposed as a noninvasive indicator of metabolic health in dairy cows, particularly during early lactation when metabolic demands are elevated. This study aimed to investigate the relationship between milk lactose levels and physiological, biochemical, and behavioral parameters in early-lactation Holstein cows. Twenty-eight clinically healthy cows were divided into two groups: Group 1 (milk lactose < 4.70%, n = 14) and Group 2 (milk lactose ≥ 4.70%, n = 14). Both groups were monitored over a 21-day period using the Brolis HerdLine in-line milk analyzer (Brolis Sensor Technology, Vilnius, Lithuania) and SmaXtec intraruminal boluses (SmaXtec Animal Care Technology^®^, Graz, Austria). Parameters including milk yield, milk composition (lactose, fat, protein, and fat-to-protein ratio), blood biomarkers, and behavior were recorded. Cows with higher milk lactose concentrations (≥4.70%) produced significantly more milk (+12.76%) and showed increased water intake (+15.44%), as well as elevated levels of urea (+21.63%), alanine aminotransferase (ALT) (+22.96%), glucose (+4.75%), magnesium (+8.25%), and iron (+13.41%) compared to cows with lower lactose concentrations (<4.70%). A moderate positive correlation was found between milk lactose and urea levels (r = 0.429, *p* < 0.01), and low but significant correlations were observed with other indicators. These findings support the use of milk lactose concentration as a practical biomarker for assessing metabolic and physiological status in dairy cows, and highlight the value of integrating real-time monitoring technologies in precision livestock management.

## 1. Introduction

For dairy cows, the early-lactation period is critical, involving intense metabolic adaptations, immune challenges, and changes in feeding behavior [1]. Understanding these complex physiological adjustments is essential for developing effective management strategies aimed at improving animal health and productivity [2].

Reliable biomarkers that reflect the cow’s physiological and metabolic status play a key role in herd management [3]. Among various tools, in-line milk analyzers such as the Brolis HerdLine system (Brolis Sensor Technology, Vilnius, Lithuania) have gained prominence for continuously measuring milk components, including lactose, fat, and protein, as well as calculating the fat-to-protein ratio [1]. As precision farming technologies advance, these systems are expected to integrate additional data sources and further improve health monitoring and decision-making on farms [2]. The Brolis HerdLine milk analyzer, for example, utilizes advanced spectroscopic techniques to assess important milk traits, including lactose concentration and fat-to-protein ratio, enabling real-time health assessments during critical periods such as early lactation [4]. Several studies have demonstrated associations between milk composition and metabolic or inflammatory conditions, underlining the value of in-line monitoring for early disease detection and improved welfare [5,6].

Lactose concentration in milk is an important indicator of metabolic health, as it is closely related to energy balance. Cows experiencing negative energy balance (NEB) often show reduced lactose levels [7]. Additionally, high levels of non-esterified fatty acids (NEFAs) and β-hydroxybutyrate (BHBA), which signal ketosis, have been linked to lower lactose concentrations [8]. Lactose is also considered a useful marker for udder health, particularly in cases of mastitis [9]. A lactose threshold of 4.70% was selected for this study, based on evidence showing that cows with levels below this value are more likely to experience metabolic disturbances, including subclinical ketosis, negative energy balance, and elevated somatic cell counts [8,9]. By contrast, cows with lactose levels above 4.70% tend to have better physiological performance, higher milk yield, more stable liver and kidney function, and better behavioral parameters such as higher rumination and water intake [8,9,10]. Moreover, higher lactose concentrations have been linked to improved reproductive outcomes, making this trait valuable for breeding programs focused on overall health and productivity [11].

Regularly monitoring milk lactose enables early identification of subclinical health issues, allowing farmers to intervene proactively and minimize production losses [12]. Combining milk lactose data with advanced monitoring tools like the SmaXtec bolus system offers a more comprehensive overview of each cow’s metabolic and reproductive status [12,13]. The SmaXtec bolus continuously monitors parameters such as activity, reticulorumen pH, and temperature, and its data have been found to correlate with milk lactose fluctuations and behavioral patterns like rumination and drinking behavior [14]. Integrating these data supports timely nutritional or management adjustments when deviations in lactose levels are detected [12].

Understanding the interplay between blood biochemical markers and milk lactose levels is also crucial, as shifts in blood metabolites can directly impact milk composition [9]. Combined monitoring of blood and milk parameters enables more targeted interventions, helping improve animal health and overall herd productivity [8,15].

Based on the hypothesis that milk lactose concentration serves as a noninvasive indicator of metabolic and physiological status in early lactation, this study investigated its association with blood biochemical traits, milk composition, and behavioral parameters. We aimed to utilize real-time data from intraruminal sensors and in-line milk analyzers to provide a comprehensive picture of cow health during this demanding period.

## 2. Materials and Methods

### 2.1. Study Animal Housing Conditions

This research was carried out at a Lithuanian university’s Practical Training and Research Center and large animal clinic, located in central Lithuania in Eastern Europe. The experiment commenced on 7 February 2025 and concluded on 27 February 2025. Holstein dairy cows were chosen for this study. In this study, we selected 28 Holstein cows from a total of 109, comprising 17 multiparous and 11 primiparous individuals, for comprehensive monitoring. The cows ranged from 9 to 59 days in milk (DIM), with a mean of 28 DIM. Only animals in good health, as determined by thorough clinical evaluations, were included. All cows were assessed by the same veterinarian following a standardized clinical examination protocol. A comprehensive physical examination was performed on each cow to verify its general health and rule out any systemic diseases or incapacitating conditions. All of the chosen cows were found to be healthy and free of any clinical signs of disease, according to the clinical examinations. The cows were milked utilizing DeLaval milking robots (DeLaval Inc., Tumba, Sweden). The mean body weight of the cows was 540 ± 45 kg. They were accommodated in ventilated free-stall barns. In 2024, the mean milk yield (4.1% fat, 3.4% protein) was 10,304 kg per cow annually. The animals were housed in a loose system and provided with a total mixed ration (TMR) throughout the year, designed to fulfill their physiological requirements. Feeding occurred daily at 08:00 and 16:00. Cows had unlimited access to drinking water. The TMR composition is detailed in Table 1 and Table 2.

### 2.2. Parameter Registration

The Brolis HerdLine in-line milk analyzer (Brolis Sensor Technology, Vilnius, Lithuania) was used in this study to record the composition of milk, and the SmaXtec (SmaXtec animal care GmbH, Graz, Austria) was used to track the cows’ activity behaviors, water intake, reticulorumen temperature, and rumination time. In 2025, blood samples were drawn from the coccygeal vein five times a week at regular intervals on February 7, 12, 17, 22, and 27.

### 2.3. Milk Parameters

Each cow’s daily lactose concentration, milk fat, protein levels, and fat-to-protein ratio were recorded using an in-line milk analyzer created by Brolis Sensor Technology (Vilnius, Lithuania). This instrument makes use of a specially made external cavity laser spectrometer based on GaSb, which operates in the 2100–2400 nm spectral range and is widely tunable. It continuously tracked the milk’s flow in transmission mode during the milking process. The gathered molecular absorption spectra were analyzed to determine the concentrations of important milk constituents, such as fat, protein, and lactose. This small “mini spectroscope” could be installed straight onto milking stalls or robotic milking systems, eliminating the need for extra chemicals or upkeep. Every five seconds during each milking, the composition of the milk was measured. Based on the dynamics of milk flow, weighted averages of fat, protein, and lactose were computed to get the final values, which represented the complete milking session.

### 2.4. Blood Parameters

Two hours after each cow was fed, blood samples were taken. Every sample was collected throughout the clinical examination. We headlocked the cows each time we took a blood sample from the coccygeal vein using a needle syringe. To analyze the blood’s biochemical profile, blood samples were drawn from the coccygeal vein using an evacuated tube devoid of anticoagulant (BD Vacutainer^®^, Eysin, Switzerland). For additional analysis, the blood samples were sent to the Laboratory of Clinical Tests at the Large Animal Clinic of the Veterinary Academy, Lithuanian University of Health Sciences. Blood samples were then centrifuged at 1500× *g* for 15 min in the lab.

The concentrations of non-esterified fatty acids (NEFAs), albumin (ALB), calcium (Ca), C-reactive protein (CRP), iron (Fe), glucose (GLUC), magnesium (Mg), triglycerides (TRIG), and urea (UREA), as well as the enzymatic activities of alkaline phosphatase (ALP), alanine aminotransferase (ALT), aspartate aminotransferase (AST), and gamma-glutamyl transferase (GGT), were measured using an automated wet chemistry analyzer (RX Daytona, Randox Laboratories Ltd., London, UK) with dedicated Randox clinical chemistry reagent kits.

### 2.5. Monitoring of Cow Behavior and Physiological Parameters

Each of the 28 cows in the trial was given an oral SmaXtec bolus (SmaXtec animal care technology^®^, Graz, Austria) at the start of the experiment. As directed by the manufacturer, the boluses were inserted into the reticulorumen using specialized applicator equipment. Cows were kept in self-locking head gates during administration, and their heads were gently held in position so that the bolus could be placed near the base of the tongue. Two hours after injection, all cows were observed to look for any negative reactions.

SmaXtec boluses were used to continuously monitor reticulorumen parameters, including temperature, rumination time, physical activity, and water intake. Prior to administration, each bolus was activated, paired with the respective cow by matching it to the ear tag number, and connected to the base station to enable real-time data transmission. The boluses recorded data at 10 min intervals throughout the experiment. Data were collected via antennas connected to the SmaXtec system, and stored on an internal memory chip controlled by a microprocessor and analog-to-digital converter. All information was compiled and managed using SmaXtec Messenger^®^ software (version 4).

### 2.6. Group Creation

According to the literature [8] and the level of lactose in milk, 28 cows were categorized into two groups: group 1—milk lactose < 4.70% (n = 14) (8 multiparous and 6 primiparous), and group 2—milk lactose ≥ 4.70% (n = 14) (9 multiparous and 5 primiparous). Each group contained cows that were primiparous (n = 11) and multiparous (n = 17). Grouping was performed prior to data collection and remained consistent throughout the study period.

### 2.7. Statistical Analysis

For statistical data analysis, SPSS 25.0 (SPSS Inc., Chicago, IL, USA) was utilized. The normality of the data distribution was assessed using the Shapiro–Wilk test. The mean ± standard error of the mean was used to express the results. The linear association between the examined qualities was found using the Pearson correlation. To compare the mean values of the investigated traits between the two lactose groups, multiple independent samples t-tests were performed. A *p*-value of less than 0.05 was considered statistically significant.

## 3. Results

### 3.1. Examined Characteristics Based on the Lactose Group

The milk yield in the second lactose group (≥4.70%) was significantly higher, showing a 12.76% increase compared to the first lactose group (<4.70%) (*p* < 0.05). The fat content was 0.39% higher in the first lactose group compared to the second group (*p* < 0.01). Water intake was significantly higher in the second lactose group, with a 15.44% increase compared to the first group (*p* < 0.01). ALT levels were 22.96% higher in the second lactose group compared to the first group (*p* < 0.001). The analysis of Fe levels revealed a 13.41% higher mean difference in the second lactose group compared to the first group (*p* < 0.05). The GLUC levels were 4.75% higher in the second lactose group compared to the first group (*p* < 0.05). Mg levels were 8.25% higher in the second lactose group compared to the first group (*p* < 0.05). UREA levels were 21.63% higher in the second lactose group compared to the first group (*p* < 0.001) (Table 3). No statistically significant average differences were detected between the lactose groups for other traits investigated.

### 3.2. Correlation Between Behavioral, Milk, and Blood Parameters

**Figure 1:**
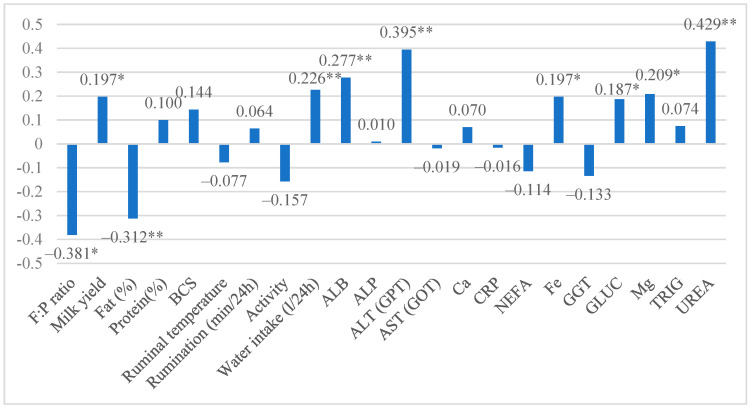
Relationship between lactose and investigated traits. * indicates a significant difference from the other lactose group (**—*p* < 0.01, *—*p* < 0.05).

Data analysis revealed a low to moderate relationship between the lactose groups and the investigated traits. A moderate positive correlation was detected between the lactose groups and urea levels (r = 0.429, *p* < 0.01). Also, a low positive statistically significant relationship was found with ALT, ALB, and water intake (*p* < 0.01) and with milk yield, Fe, GLUC, and Mg (*p* < 0.05). A low negative relationship was detected between lactose and F:*p* ratio (r = −0.381) and with fat content (r = −0.312, *p* < 0.01) (Figure 1).

## 4. Discussion

Lactose concentration in milk emerged as a potential indicator of metabolic health and activity patterns in early-lactation dairy cows, reflecting the animals’ energy balance and overall physiological status. This study demonstrated that cows in the second lactose group (milk lactose ≥ 4.70%) produced significantly more milk, with a 12.76% increase compared to those in the first group (milk lactose < 4.70%), while the fat content was 0.39% higher in the first lactose group than in the second. This difference in production is further supported by a low but statistically significant positive correlation with milk yield. Several other studies have investigated the link between milk lactose content and yield in dairy cows: one such study found that cows with higher in-line milk lactose concentrations (≥4.70%) produced significantly more milk, yielding 16.14% more than cows with lower lactose levels [8]. Antanaitis et. al. [12] also reported a positive correlation between milk lactose percentage and milk yield, suggesting that cows producing milk with higher lactose content generally exhibit greater milk production. Lactose production in the mammary gland plays a key role in determining milk volume, functioning as an osmotic regulator that draws water into the milk. During lactation, approximately 20% of circulating blood glucose is used for lactose synthesis, highlighting the significant energy investment required for this process. Consequently, higher lactose concentrations are generally associated with greater milk yield, which increases the cow’s demand for water to support milk formation [16]. This physiological relationship was reflected in our findings, where cows in the second lactose group (milk lactose > 4.70%) exhibited significantly higher water intake—15.44% more than those in the first group (*p* < 0.01). Costa et al. [16] highlighted that lactose synthesis constitutes a major metabolic process in dairy cows, placing considerable demand on hepatic glucose production through gluconeogenesis. This metabolic burden may contribute to the observed 22.96% increase in ALT levels (*p* < 0.001) in cows with higher milk lactose concentrations, suggesting enhanced hepatic activity associated with elevated lactose output, further supported by a low but statistically significant positive correlation with ALT. A study conducted by other researchers reported that changes in blood metabolites were strongly associated with milk production traits, including lactose content. Notably, higher milk lactose levels showed a positive correlation with elevated serum albumin concentrations, indicating a potential connection between lactose synthesis and protein metabolism in dairy cows [17]. Consistent with these findings, our research also revealed a low but statistically significant positive correlation with ALB. We also found that cows in the higher lactose group showed a 13.41% increase in serum iron concentrations compared to those in the lower lactose group (*p* < 0.05), and this difference was further supported by a low but statistically significant positive correlation with Fe. This finding may reflect increased systemic iron demand associated with higher metabolic activity and oxygen transport needs. As noted by Antanaitis et al. [12], higher milk lactose concentrations are positively associated with increased milk yield, a process that requires enhanced metabolic function and adequate iron availability to support tissue oxygenation and enzymatic activity during peak lactation. The present findings revealed that cows in the second lactose group had 4.75% higher blood glucose levels compared to the first group (*p* < 0.05), which aligns with previous studies demonstrating a positive correlation between blood glucose concentration and milk lactose content [8], and is further supported by a low but statistically significant positive correlation with GLUC. This can be explained by the fact that glucose is essential for lactose production in the mammary gland; approximately 20% of circulating blood glucose is converted into lactose during lactation, underscoring the substantial energy investment required for this process [16]. Serum Mg concentrations were 8.25% higher in the second lactose group compared to the first (*p* < 0.05), reflecting a significant variation in mineral status between groups, which was further supported by a low but statistically significant positive correlation with Mg. Magnesium is essential for numerous metabolic functions, particularly as a cofactor in enzymatic processes related to ATP synthesis and carbohydrate metabolism. Elevated magnesium levels in cows with higher milk lactose concentrations may support more efficient lactose synthesis, likely due to improved energy metabolism and mineral balance in these higher-performing animals [18]. Moreover, urea concentrations were 21.63% greater in the second lactose group (*p* < 0.001), suggesting differences in protein metabolism, which is further supported by a moderate positive correlation between lactose groups and urea levels (r = 0.429, *p* < 0.01). As a byproduct of nitrogen metabolism, blood urea levels are influenced by dietary protein intake, energy availability, and hepatic function. Higher urea levels in the second group may indicate increased protein intake or utilization, consistent with the elevated metabolic demands of enhanced milk and lactose production [12,19].

Despite valuable insights, this research has certain limitations regarding the interpretation of milk lactose concentration in relation to genetic factors and parity. The genetic influence on lactose concentration is relatively weak compared to other milk components like fat and protein. While breed differences do exist—for example, Holstein cows typically show slightly higher lactose levels than Jerseys—the within-breed genetic variability is limited, making it a less useful trait for selection or prediction [20]. The role of parity also remains ambiguous; although primiparous cows sometimes show marginally higher lactose levels than multiparous cows, such differences are often confounded by health status, energy balance, or stage of lactation [21]. Future studies should increase sample sizes and include a wider genetic base and diverse parity groups to improve generalizability and clarify these subtle effects. In addition, short monitoring periods may fail to capture dynamic changes in lactose levels throughout lactation. Extending the monitoring duration and employing more sensitive diagnostic tools could improve the detection of minor yet meaningful trends, allowing for more precise use of lactose as a biomarker in dairy herd management.

## 5. Conclusions

The results of this study suggest that milk lactose concentration may be a valuable, noninvasive indicator reflecting the metabolic and physiological status of dairy cows during early lactation. We observed that higher lactose levels were associated with greater milk yield and higher concentrations of glucose, urea, alanine aminotransferase, magnesium, and iron, which may point to better overall metabolic activity and liver function. However, it is important to emphasize that these findings describe associations rather than direct causal effects. Despite this, monitoring milk lactose could offer practical benefits for farmers by helping identify cows at risk of metabolic imbalances early and supporting more targeted herd management decisions.

## Figures and Tables

**Table 1:** Composition of TMR *.

TMR Component	Value
Corn silage	31%
Alfalfa grass hay	4%
Grass silage	10%
Grain concentrate mash	49%
Mineral mix	6%

* TMR—total mixed ration.

**Table 2:** Chemical composition of TMR *.

TMR Component	Value
Dry Matter (DM)	50.7%
Neutral Detergent Fiber (NDF)	28.3% of DM
Acid Detergent Fiber (ADF)	19.8% of DM
Non-Fiber Carbohydrates (NFC)	38.7% of DM
Crude Protein (CP)	15.8% of DM
Net Lactation Energy	1.6 Mcal/kg

* TMR—total mixed ration.

**Table 3:** Investigated traits according to lactose group.

Investigated Trait	Lactose Group	(M ± SEM)
Milk yield, kg	1	33.54 ± 1.340 *
	2	37.82 ± 1.011
Fat %	1	4.42 ± 0.098 ***
	2	4.03 ± 0.052
Protein %	1	3.33 ± 0.062
	2	3.39 ± 0.021
Body condition score (BCS)	1	3.33 ± 0.064
	2	3.44 ± 0.032
Ruminal temperature	1	38.63 ± 0.060
	2	38.58 ± 0.028
Rumination (min/24 h)	1	506.22 ± 10.535
	2	514.44 ± 5.528
Activity	1	5.96 ± 0.290
	2	5.34 ± 0.176
Water intake	1	112.81 ± 6.593 **
	2	130.23 ± 3.141
Albumin (ALB)	1	34.34 ± 0.538
	2	36.27 ± 0.294
Alkaline phosphatase (ALP)	1	49.27 ± 2.576
	2	49.64 ± 1.800
Alanine aminotransferase (ALT)	1	22.21 ± 0.879 ***
	2	27.31 ± 0.537
Aspartate aminotransferase (AST)	1	86.82 ± 3.329
	2	85.96 ± 2.103
Calcium (Ca)	1	2.46 ± 0.036
	2	2.48 ± 0.017
C-reactive protein (CRP)	1	10.49 ± 0.804
	2	10.30 ± 0.555
Non-esterified fatty acids (NEFA)	1	0.30 ± 0.035
	2	0.25 ± 0.016
Iron (Fe)	1	17.75 ± 0.934 *
	2	20.13 ± 0.526
Gamma-glutamyl transferase (GGT)	1	29.99 ± 1.194
	2	28.01 ± 0.643
Glucose (GLUC)	1	3.37 ± 0.065 *
	2	3.53 ± 0.038
Magnesium (Mg)	1	0.97 ± 0.032 *
	2	1.05 ± 0.017
Triglycerides (TRIG)	1	0.09 ± 0.004
	2	0.10 ± 0.011
UREA	1	3.56 ± 0.101 ***
	2	4.33 ± 0.078

Values are presented as mean ± SEM. Group 1: lactose < 4.70%; Group 2: lactose ≥ 4.70%. * indicates a significant difference from the other lactose group (***—*p* < 0.001, **—*p* < 0.01, *—*p* < 0.05).

## Data Availability

Data are contained within the article.
